# The Management of Cases of Cholera Morbus

**Published:** 1877-04-20

**Authors:** Charles C. Pike

**Affiliations:** Peabody, Mass.


### THE MANAGEMENT OF CASES OF CHOLERA MORBUS.

By CHARLES C. PIKE, M. D., of Peabody, Mass.

Every active physician is aware how unpleasant it is to be called out of a sound sleep in the middle of the night, as we usually are, to relieve a case of acute indigestion or cholera morbus, as it is commonly called. He finds his patient suffering terribly with pain in the stomach and bowels, vomiting and purging, the air in the room necessarily rendered sickening and disgusting thereby; or, perhaps not having been called early enough, he finds the patient rapidly sinking into a collapsed state, with cold extremities, cramps, etc. Writers on this disease advise giving small doses of morphine, morphine and calomel, brandy, and ice, etc. All of which, in my experience, are very likely to be rejected by the stomach, and generally we are obliged to work over our patient for an hour, and oftener two or three hours, before we can feel sure that the disease is controlled. The hypodermic use of morphine will usually give prompt relief; but from prejudice on the part of friends, or from other causes, we are often debarred from using it.

Living in a town where there are a large number of Irish laborers, and consequently having many cases of cholera morbus to treat, I have adopted a plan of treatment which, while I by no means claim it as original, serves me admirably; *i. e.*, I do not now find myself obliged to remain with the patient but a few moments, where formerly I was obliged to stay an hour or two. Immediately upon reaching the bedside of the cholera morbus patient, I order "saleratus and water," viz., half-teaspoonful of saleratus, to a cup-full of water of summer temperature. If the patient has already cleared the stomach by vomiting, this will at once neutralize the sour condition of that organ, and relieve the nausea, and if vomiting have not taken place, the combination of the alkali with the acid contents of the stomach will usually induce action enough to cause vomiting, which of course is desirable to an extent of unloading the stomach of its contents, after which I give more of the saleratus water, which will soon stop the vomiting. Then, with a small 2-oz. metallic syringe, which I always carry—one with a ring on the end of the piston, so that it can be used with one hand, is the best—I use a small clyster composed of from one-half to one teaspoonful of laudanum, of which I always carry a small phial, and enough of the saleratus water to nearly or quite fill the syringe; this to be gently thrown into the rectum by yourself, or the nurse, if you can depend upon her doing it faithfully; tell them if the patient vomits to give the saleratus water, of course, not omitting to apply external warmth or irritants, as may be necessary, and then go back to your bed again, seldom staying in the house more than twenty minutes.

I have treated a great many cases in this way—a hundred at least—and as yet have never been called to the same patient twice in the same night, an event which happened far too often when I used other means for controlling the disease. There is no dread, lest by mistake on the part of attendants, an overdose of opiate may be administered. The laudanum acts as a sedative and also a stimulant, just what the patient needs, and the dose, from a half to one teaspoonful of laudanum, by clyster, would not be likely, under any circumstances, to do harm.—[Med. and Surg. Rep.

